# Ion Channel Lateral Diffusion Reveals the Maturation Process of the Neuronal Actin Cytoskeleton

**DOI:** 10.1093/function/zqad029

**Published:** 2023-06-06

**Authors:** Luis A Pardo

**Affiliations:** Max-Planck Institute for Multidisciplinary Sciences, Oncophysiology Group, City Campus, Hermann-Rein-Str. 3, 37075 Göttingen, Germany

## A Perspective on “Differential Control of Small-conductance Calcium-activated Potassium Channel Diffusion by Actin in Different Neuronal Subcompartments”

Small-conductance calcium-activated potassium (SK) channels are voltage-independent K^+^ channels that activate in response to a rise in cytoplasmic Ca^2+^^1^. In neurons, they are, therefore, able to reduce Ca^2+^ entry in spines and dendrites, limiting prolonged depolarization. SK channels can also be found in the soma and in axons, where they likely contribute to spike frequency adaptation. Gu and colleagues used single-particle tracking^2^ of SK channels in different areas of pyramidal hippocampal neurons in cultures of different ages. The diffusion coefficient of SK channels was determined along the maturation process of the neuronal culture using biotinylated apamin and streptavidin-conjugated quantum dots to label the channels in combination with total internal reflection microscopy. At the same time, actin cytoskeleton integrity was manipulated pharmacologically to investigate its impact on the diffusion velocity of SK channels. The approach allowed tracking the diffusion of the channel in different compartments, which served as a proxy to determine the stability of actin cytoskeletal structures. Since stable actin filaments limited the distribution of SK channels, it was possible to infer how structured the actin cytoskeleton was along the maturation process. Importantly, the submembrane actin cytoskeleton is increasingly regarded as a crucial factor for modulating the activity of ion channels and transporters at the plasma membrane (see Morachevskaya and Sudarikova^3^).

Neuronal function critically depends on the cytoarchitecture of the neuron. The cytoskeleton plays a critical role in maintaining the proper neural computation that goes far beyond mere mechanical stability and shape maintenance. Specialized structures crucial for neurotransmission, such as the node of Ranvier, the axon initial segment (AIS), or synaptic terminals, are maintained and dynamically modulated by cytoskeletal interactions. The knowledge of such modulatory roles has advanced markedly in the last decade. For example, the AIS attracts attention from the structural, molecular, cell biological, and computational points of view. The organization of the AIS with periodic rings of actin separated by spectrin tetramers fixed to the microtubules and the plasma membrane through ankyrin^4^ offers a scaffold for tethering and concentrating ion channels. The high density of Na^+^ channels, if not necessary for the initiation of the AP, is required for temporal accuracy and therefore determines the bandwidth of excitation. It is therefore crucial for all forms of neuronal computing.^5^ Voltage-gated sodium and potassium channels, as workhorses of the action potential, are positioned precisely through the organization of the AIS, and their distribution has been thoroughly characterized (see Leterrier^6^ and references therein). Sodium and certain potassium channels (Kv7.2/Kv7.3) bind to ankyrin and are therefore located in the repeats where the relative binding affinities to ankyrin determine the proportion between Na and K permeabilities^7^ and consequently shape the action potential. In contrast, other potassium channels (Kv1.1 and Kv1.2) are excluded from these structures and therefore interact closely with actin-based structures. The interactions with actin are also more complex than initially thought. For example, inactive or “reserve” Kv2 channels are clustered in actin-dependent structures called corrals; however, this is not a mere physical restriction, but determines the formation of endoplasmic reticulum–plasma membrane contacts that in turn is crucial for Ca^2+^ signaling.^8^ However, less is known about other ion channels relevant to the modulation of signaling. For example, the localization of voltage-gated Ca^2+^ channels gives rise to distinct Ca^2+^ entry microdomains, close to intracellular stores or more diffuse along the AIS^9^ depending on the molecular identity of the Ca^2+^ channel.

The results of the study of the diffusion of SK channels in developing neurons allowed us to determine the gradual increase in complexity of the submembrane skeleton in the soma and dendrites of neurons up to approximately 10 d *in vitr*o (DIV), with minor changes after that time point. Disruption of actin filaments increased the diffusion velocity of channels, indicating that actin cytoskeleton is the major player in fixing the distribution of SK channels. In contrast, in the AIS, SK channels were immobilized at an earlier time point that did not match the formation of the characteristic periodic actin rings. Importantly, disruption of the actin cytoskeleton increased the mobility of SK channels at early time points when the AIS is morphologically established but failed to do so after 14 DIV, in agreement with the described robustness of the mature AIS, which once established, is maintained regardless of disruption of microtubules.^10^

In summary, although the study of ion channel lateral diffusion in neurons (and non-excitable cells) is important on its own, it also opens the way to detailed studies on the formation and maturation of cytoskeletal-dependent complexes like the AIS and, therefore, the development of mature signal integration in neurons.
